# Risk and protective factors associated with depressive symptoms among school-going adolescents in Malaysia: a cross‑sectional study

**DOI:** 10.1186/s12888-025-07319-9

**Published:** 2025-09-30

**Authors:** Norhafizah Sahril, Hamizatul Akmal Abd Hamid, Mohamad Aznuddin Abd Razak, Sheikh Shafizal Sheikh Ilman, Filza Noor Ansari, Noor Ani Ahmad

**Affiliations:** 1 Institute for Public Health, National Institutes of Health, Ministry of Health Malaysia, Block B5, No 1, Jalan Setia Murni U13/52, Seksyen U13, Setia Alam, Shah Alam, Selangor 40170 Malaysia; 2 Institute for Health and Behavioural Research, National Institutes of Health, Ministry of Health, Shah Alam, Selangor Malaysia

**Keywords:** Depressive symptoms, Adolescents, NHMS, Mental health

## Abstract

**Introduction:**

Depression is a growing global concern, particularly among Malaysian adolescents, where its prevalence is on the rise. This study investigates both risk and protective correlates of depressive symptoms in a representative sample of school-attending Malaysian adolescents.

**Methods:**

Data from the 2022 National Health and Morbidity Survey (NHMS): Adolescent Health Survey, a comprehensive nationwide study. A multistage stratified cluster sampling method was used, and it involved two stages. The survey included a representative sample of secondary school students. Depressive symptoms were assessed using the Patient Health Questionnaire (PHQ-9), where a score of 10 or above indicated have depressive symptoms. Descriptive and complex sample logistic regression analyses were conducted using SPSS version 26.0.

**Results:**

The study involved 33,523 school-going adolescents, revealing a depressive symptoms prevalence of 26.9%. Multiple logistic regression analysis uncovered that female adolescents and those in higher age were more susceptible to depressive symptoms. In contrast, Chinese and Indian adolescents showed lower susceptibility to depressive symptoms. Factors such as parental separation, widowhood, loneliness, lack of close friendships, unable to sleep due to worry, bullying, truancy, and deficient parental bonding or connectedness were associated with a higher likelihood of depressive symptoms.

**Conclusions:**

Depressive symptoms is a significant issue among Malaysian school-going adolescents, with a prevalence rate of one in four (26.9%). Female adolescents and older students were found to have significantly higher odds of experiencing depressive symptoms, while adolescents of Chinese and Indian ethnicity were less likely to be affected. Other notable correlates included parental separation or widowhood, loneliness, lack of close friends, difficulty sleeping due to worry, bullying, truancy, and poor parental bonding and connectedness. Thus, it is crucial to develop targeted prevention and intervention programs for at-risk adolescents to enhance their mental health and well-being. The multiple logistic regression analysis showed that.

## Introduction

Adolescence is marked by rapid physiological, emotional, and cognitive changes, which heighten susceptibility to psychological challenges such as depression. During this stage, depression stands out as one of the most prevalent and serious mental health concerns globally [1]. It may manifest as persistent sadness, fatigue, disrupted sleep, low self-esteem, and, in severe cases, suicidal ideation among adolescents [2, 3].

Globally, the estimated prevalence of adolescent depression varies considerably. A general estimate suggests that approximately 7.4% of adolescents experience depression [4]. However, figures differ depending on data sources, age groups, and assessment methods. For instance, the Global Health Data Exchange (GHDx) reports that 1.4% of adolescents aged 10–14 and 3.5% of those aged 15–19 are affected [5]. Such differences may stem from varying definitions of depression, screening tools, cultural perceptions, and reporting practices. These inconsistencies underscore the importance of contextualizing prevalence data when making cross-national comparisons.

Regional studies across Asia also reveal wide variations in the prevalence of depressive symptoms, for example, 49.2% in India [6], 17.2% in Pakistan [1], 36% in Sri Lanka [7], and 52.6% in Iran [8]. In Malaysia, the latest findings from the NHMS 2023 indicate a reported prevalence of 7.9% for depressive symptoms specifically among adolescents aged 16 to 19 years. This figure is derived from a national household survey that encompassed the Malaysian population across all age groups [9]. However, methodological differences, regional sampling, and inconsistent definitions of depressive symptoms may contribute to these disparities [10]. This highlights the need for more comprehensive and nationally representative data to better understand the burden and determinants of adolescent depression.

Previous research has identified several factors associated with adolescent depression, including sociodemographic variables (e.g., gender, age, family structure), interpersonal issues (e.g., peer bullying, loneliness, poor parental bonding), and behavioral aspects (e.g., school absenteeism, sleep problems) [11–16]. While these studies provide important insights, many have notable limitations. Some are limited to urban or single-state samples [17], while others primarily focus on risk factors without considering protective elements, such as supportive relationships and positive social environments [18, 19]. Furthermore, few studies adopt a holistic framework that integrates both individual and contextual factors when examining depressive symptoms in adolescents [20].

Despite increasing awareness of adolescent mental health issues, Malaysia still lacks recent, large-scale, school-based investigations that offer a nationally representative understanding of the factors influencing depression. Given Malaysia’s unique socio-cultural diversity, rapid modernization, and growing concerns around youth well-being, there is an urgent need to explore how these dynamics impact adolescent mental health outcomes [21]. In addition, identifying both risk and protective factors is essential for informing school-based interventions and national mental health strategies [22, 23].

Therefore, this study aims to provide a thorough understanding of the factors affecting depression among Malaysian school-going adolescents aged 13 to 17, using the most recent national school-based data. By identifying both risk and protective factors, this research seeks to address current gaps in the literature and contribute to the development of evidence-based policies and interventions tailored to the Malaysian context.

## Methodology

### Study design, sample size and sampling design

The National Health and Morbidity Survey (NHMS) serves as a key platform for monitoring the health status of Malaysia’s population. Its primary objectives are to provide community-based data on common health issues, health service utilization, and healthcare expenditures. The findings from the NHMS assist the Ministry of Health in reviewing health program priorities and activities, planning resource allocation, and evaluating the effectiveness of implemented strategies. Since 2011, the NHMS has been conducted annually, with the Ministry of Health selecting the focus areas each year. This study uses data from the 2022 NHMS: Adolescent Health Survey, which involved a cross-sectional analysis of secondary school students aged 13 to 17 years across Malaysia. The survey encompassed all government and private schools overseen by the Ministry of Education (MOE) and Majlis Amanah Rakyat (MARA) or the Council of Trust for the People. However, it excluded special education schools and those unable to provide consent or assent to participate. This survey took place between June and July 2022. The sample size was calculated to ensure the statistical reliability and representativeness of the findings, while adhering to budget constraints. The sample size was determined based on the objectives of 10 core modules and 1 additional module, which covered topics such as alcohol use, dietary behaviours, drug use, hygiene, mental health problems, physical activity, protective factors, sexual behaviours, tobacco use, violence and unintentional injuries, as well as adolescents’ perspectives on the impact of the COVID-19 pandemic on their families. The sample size was calculated using the formula for a single proportion. Several criteria were considered, including the prevalence of the previous Adolescent Health Survey (AHS) 2017, a margin of error ranging from 0.01 to 0.05, and a confidence interval of 95%. Adjustments were made for the total number of the target population (based on the enrolment data of school-going adolescents in 2021) to ensure an optimal sample size that provided sufficient precision for estimating the prevalence of the health condition specified in the survey. These adjustments included applying a design effect of 2 and accounting for complex sampling analysis, which incorporated a non-response rate of 20%. Thus, the final sample size required for national-level was 36,000 adolescents and 2,250 adolescents for state-level analysis.

A multistage stratified cluster sampling method was employed, comprising two stages. In the first stage, a comprehensive list of eligible secondary schools across Malaysia was used to randomly select 240 schools through simple random sampling, with probability proportional to size (PPS) based on student enrolment across Forms 1 to 5. In Malaysia, school grade levels are referred to as Forms. Specifically, Form 1 corresponds to age 13, Form 2 to age 14, Form 3 to age 15, Form 4 to age 16, and Form 5 to age 17. Each state contributed 16 schools, except for the smaller federal territories of Labuan and Putrajaya, where 8 schools were selected. In the second stage, systematic probability sampling with a random starting point was used to select classes within each chosen school, covering all Forms 1 through 5. All students in the selected classes were invited to participate in the survey. To approach students, trained researchers visited classrooms, explained the purpose of the study, and provided instructions for completing the self-administered questionnaires. Participation was conditional upon obtaining written parental consent, and assent was implied through the student’s willingness to complete the questionnaire. The inclusion criteria were students aged 13 to 17 years, while those absent on data collection days or without parental consent were excluded. Detailed methods of the National School-Based Health Survey 2022 are available elsewhere [24].

### Sample weighting

The sample weight was calculated to adjust the sample data for ensuring sample representativeness in line with complex sampling analysis method. The types of sample weighting used in this study was post-stratification weighting. The weight used for sample weight estimation was given as follows:

$$\mathrm W=\mathrm W1\;\mathrm x\;\mathrm W2\;\mathrm x\;\mathrm F\;\mathrm x\;\mathrm{PW},$$

Where W1 is the inverse of the probability of selecting the school; W2 is the inverse of the probability of selecting the classroom within the school; F is the inverse of the response rate of school level, class and student; and PW is the poststratification adjustment factor based on gender and grade/form of the students.

### Measures

#### Depressive symptoms

The variable under investigation, namely depressive symptoms, was evaluated using the Patient Health Questionnaire-9 (PHQ-9). This self-report instrument consists of nine questions aligned with the nine DSM-IV criteria used to diagnose depression. It is employed to determine the presence or absence of depression. In this study, the validated Malay version of the Patient Health Questionnaire-9 (PHQ-9) was used to assess depressive symptoms. This instrument has demonstrated reliability and validity, with good internal consistency indicated by a Cronbach’s alpha of 0.70. In terms of concurrent validity, it showed a sensitivity of 87%, specificity of 82%, a positive likelihood ratio (LR) of 4.8, and a negative LR of 0.16 [25]. The original English version, developed by Kroenke, Spitzer, and Williams in 2001, served as the basis for this tool [26]. Although originally designed for adults, the PHQ-9 has since been validated in adolescent populations across multiple countries. Recent studies from China [27], Spain [28], South Africa [29], and the United States [30] have consistently demonstrated strong psychometric properties in adolescents, including high sensitivity, specificity, internal consistency, and measurement invariance. For example, a 2024 study in China confirmed the PHQ-9’s unidimensional structure and appropriateness for adolescent use [27], while a 2023 Spanish study found measurement invariance across gender among adolescents aged 12–18 [28]. Additional evidence from South Africa [29] and the United States [30] further supports its effectiveness in detecting depressive symptoms and suicide risk in youth populations. In the Malaysian context, the PHQ-9 was translated and culturally adapted into Malay [22], and its reliability among adolescents was recently reaffirmed by Yazid and Mohd Sa’ad (2024) in a school-based sample [31]. Collectively, this body of international and local evidence affirms the PHQ-9 as a psychometrically robust and culturally appropriate tool for assessing depression in Malaysian adolescents. These findings provide strong justification for its use in the current study.

The questionnaire queries respondents about symptoms they experienced in the two weeks preceding its completion. Each of the nine items provides four response options (“not at all,” “several days,” “more than half the days,” and “nearly every day”), with corresponding scores ranging from zero (indicating no occurrence) to three (indicating almost daily occurrence), resulting in a total score between zero and 27. Individuals scoring ten or higher were categorized as experiencing depressive symptoms [25, 26].

The Adolescent Health Survey 2022 utilized a self-administered questionnaire adapted from the World Health Organization’s Global School-based Student Health Survey and was available in two languages. All questionnaires were translated into Malay and then back-translated into English to ensure translation accuracy and quality. The questionnaires were subsequently field-tested, revised, finalized, and approved by the NHMS 2022 Questionnaire Review Committee [24]. For the purposes of this study, ten independent variables were included: gender; ethnicity (Malay, Chinese, Indian, Other Bumiputera, and Others); age (13–17 years old); parental marital status (married, separated/widow/widowed); loneliness; having close friends; unable to sleep due to worry; bullied; lack of parental bonding; and parental connectedness. “Loneliness” was defined as feeling lonely “most of the time” or “always” during the 12 months preceding the survey. “Having close friends” was defined as having any close friends. “Unable to sleep” was defined as responding “most of the time” or “always” to being worried about something that prevented them from sleeping at night during the past 12 months prior to the survey. In this study, “bullied” was defined as having been bullied at least once in the past 30 days. Truancy was defined as being absent from class or school without permission for at least one day in the past 30 days. Lack of parental bonding referred to parents or guardians seldom or never knowing how their children spent their free time during this period. Lack of parental connectedness was defined as parents or guardians seldom or never understanding their children’s problems and concerns over the past 30 days [24].

#### Data analysis

Descriptive statistics were utilized to summarize the characteristics of the study population. The prevalence of depressive symptoms among adolescents was analysed based on sociodemographic characteristics and other variables. To explore the association between depressive symptoms, sociodemographic factors, and other selected independent variables, both univariable and multivariable logistic regression analyses were performed. The findings are presented as crude and adjusted odds ratios (OR) with 95% confidence intervals (CI). Variables with a p-value less than 0.25 in the univariable analysis were considered for inclusion in the final multivariable model [32]. Statistical significance in the multivariable analysis was determined using a two-tailed test with a p-value threshold of less than 0.05. To ensure the appropriateness of the logistic regression model, diagnostic testing using a classification table was conducted to assess its goodness of fit. In this study, the classification table indicated an accuracy of 82.1%, which was used as part of the evaluation process. A two-way interaction analysis between all the independent variables in the model was carried out, and no significant two-way interaction was found (*P* > 0.05). The analysis of data was conducted using SPSS version 26.0 (SPSS IBM, New York, USA), taking into consideration the sample weighting and complex sampling design.

## Results

The study population consisted of 33,523 participants with a majority being female (53.8%) and the rest male (46.2%). The age distribution was relatively even across the 13 to 17 years old range, with each age group representing approximately 18.5–21.5% of the sample. Ethnically, the majority were Malay (69.0%), followed by Chinese (15.2%), Indian (4.6%), Other Bumiputeras (8.8%), and Others (2.4%). Most participants reported that their parents were married (85.3%), while 14.7% reported their parents were separated, widowed, or widowers. In terms of social and emotional well-being, 16.4% of participants reported feeling lonely, 13.1% were unable to sleep due to worry, and 8.4% had been bullied at least once in the past 30 days. A significant portion, 25.5%, reported truancy. Additionally, 66.5% felt a lack of parental bonding, and 75.3% experienced a lack of parental connectedness. Despite these challenges, a high percentage (95.9%) reported having close friends (Table 1).

**Table 1:** Sample characteristics of secondary school students aged 13 to 17 years in Malaysia (*N* = 33,523)

Variables	*n*	Percentage (%)
Sex		
Female	18,030	53.8
Male	15,493	46.2
Age		
13	7216	21.5
14	6902	20.6
15	6460	19.3
16	6756	20.2
17	6189	18.5
Ethnicity		
Malay	23,125	69.0
Chinese	5085	15.2
Indian	1556	4.6
Other Bumiputeras	2963	8.8
Others	794	2.4
Parent’s Marital Status		
Separated/Widow/Widower	4844	14.7
Married	28,070	85.3
Loneliness		
Yes	5485	16.4
No	28,032	83.6
Has close friend		
No	1389	4.1
Yes	32,125	95.9
Unable to sleep due to worry		
Yes	4380	13.1
No	29,133	86.9
Bullied		
Yes	2769	8.4
No	30,340	91.6
Lack of Parental Bonding		
Yes	21,922	66.5
No	11,056	33.5
Lack of Parental Connectedness		
Yes	24,841	75.3
No	8150	24.7
Truancy		
Yes	8412	25.5
No	24,608	74.5

The recorded prevalence of depressive symptoms among Malaysian adolescents stands at 26.9% (95% CI: 25.84, 27.96), affecting a total of 556,498 adolescents. Notably, the prevalence is significantly higher among females, reaching 36.1% (95% CI: 34.58, 37.68), in contrast to males, where it is 17.7% (95% CI: 16.69, 18.67). Furthermore, adolescents with separated, widowed, or widower parents (34.6%) exhibit a significantly higher prevalence of depressive symptoms. The prevalence of depressive symptoms is markedly higher among adolescents who experienced persistent loneliness in the past 12 months (71.4%), lacked close friends (53.5%), were unable to sleep due to worry (72.3%), faced bullying in the last 30 days (51.3%), lacked parental bonding (31.3%), lacked parental connectedness (30.3%), and were involved in truancy (35.7%) (Table 2).

**Table 2:** Prevalence of depressive symptoms by sociodemographic and selected independent variables. (*N* = 9,103)

Variables	Depressed			Non-depressed		
	*n* (%)	95% Confidence Interval		*n* (%)	95% Confidence Interval	
		Lower	Upper		Lower	Upper
Overall	9103 (26.9)	25.84	27.96	24,304 (73.1)	72.04	74.16
Sex						
Female	6421 (36.1)	34.58	37.68	11,543 (63.9)	62.32	65.42
Male	2682 (17.7)	16.69	18.67	12,761 (82.3)	81.33	83.31
Age						
13	1704 (22.6)	21.18	24.02	5473 (77.4)	75.98	78.82
14	1882 (27.2)	25.58	28.83	4994 (72.8)	71.17	74.42
15	1802 (27.0)	25.08	28.94	4639 (73.0)	71.06	74.92
16	1882 (28.4)	26.61	30.16	4853 (71.6)	69.84	73.39
17	1833 (30.1)	28.22	31.95	4345 (69.9)	68.05	71.78
Ethnicity						
Malay	6504 (28.7)	27.46	29.89	16,544 (71.3)	70.11	72.54
Chinese	1065 (20.6)	18.31	23.05	3999 (79.4)	76.95	81.69
Indian	328 (20.2)	17.59	23.04	1220 (79.8)	76.96	82.41
Other Bumiputeras	977 (30.5)	27.07	34.13	1981(69.5)	65.87	72.93
Others	229 (28.6)	23.44	34.38	560 (71.4)	65.62	76.56
Parent’s Marital Status						
Separated/Widow/Widower	1714 (34.6)	32.81	36.45	3111 (65.4)	63.55	67.19
Married	7217 (25.6)	24.48	26.65	20,761 (74.4)	73.35	75.52
Loneliness						
Yes	3890 (71.4)	69.67	72.98	1572 (28.6)	27.02	30.33
No	5213 (18.3)	17.46	19.15	22,728 (81.7)	80.85	82.54
Has close friend						
No	740 (53.5)	50.11	56.83	647 (46.5)	43.17	49.89
Yes	8359 (25.7)	24.66	26.80	23,653 (74.3)	73.20	75.34
Unable to sleep due to worry						
Yes	3197 (72.3)	70.21	74.25	1165 (27.7)	25.75	29.79
No	5905 (20.2)	19.34	21.05	23,132 (79.8)	78.95	80.66
Bullied						
Yes	1421 (51.3)	48.40	54.20	1332 (48.7)	45.80	51.60
No	7581 (24.6)	23.60	25.61	22,668 (75.4)	74.39	76.40
Lack of Parental Bonding						
Yes	6952 (31.3)	30.04	32.54	14,896 (68.7)	67.46	69.96
No	2019 (18.1)	17.07	19.27	9006 (81.9)	80.73	82.93
Lack of Parental Connectedness						
Yes	7649 (30.3)	29.12	31.50	17,112 (69.7)	68.50	70.88
No	1322 (16.2)	14.88	17.59	6802 (83.8)	82.41	85.12
Truancy						
Yes	2970 (35.7)	34.14	37.35	5416 (64.3)	62.65	65.86
No	6008 (23.8)	22.76	24.94	18,520 (76.2)	75.06	77.24

The multiple logistic regression analysis yielded significant findings. Higher odds of experiencing depressive symptoms were observed among female adolescents (AOR: 2.39, 95% CI: 2.19, 2.61) and those in higher aged. Conversely, Chinese (AOR: 0.76, 95% CI: 0.63, 0.92) and Indian (AOR: 0.62, 95% CI: 0.51, 0.75) adolescents were less likely to experience depressive symptoms. Adolescents with parents who were separated, widowed, or divorced had higher odds of reporting depressive symptoms (AOR: 1.19, 95% CI: 1.08, 1.32). Furthermore, factors such as persistent loneliness in the past 12 months (AOR: 6.52, 95% CI: 5.90, 7.21), lacking close friends (AOR: 2.05, 95% CI: 1.70, 2.47), unable to sleep due to worry, (AOR: 5.93, 95% CI: 5.26, 6.67), current school bullying (AOR: 2.85, 95% CI: 2.46, 3.30), and involvement in truancy (AOR: 1.43, 95% CI: 1.31, 1.56) were significantly associated with depressive symptoms. Additionally, within the past 30 days, factors such as a lack of parent or guardian bonding (AOR: 1.40, 95% CI: 1.27, 1.55) and connectedness (AOR: 1.58, 95% CI: 1.39, 1.79) were also found to be significantly associated with depressive symptoms (Table 3).

**Table 3:** Factor associated with depressive symptoms among School-going adolescents in Malaysia

Variables	Crude OR			*p*-value	Adjusted OR			*p*-value
	Exp(B)	95% Confidence Interval			Exp(B)	95% Confidence Interval		
		Lower	Upper			Lower	Upper	
Gender								
Male	1.000				1.000			
Female	2.636	2.45	2.84	< 0.001	2.394	2.19	2.61	< 0.001
Age								
13	1.000				1.000			
14	1.280	1.17	1.42	< 0.001	1.210	1.08	1.36	0.001
15	1.267	1.13	1.42	< 0.001	1.139	1.01	1.29	0.038
16	1.358	1.22	1.51	< 0.001	1.191	1.06	1.34	0.004
17	1.474	1.32	1.64	< 0.001	1.305	1.14	1.49	< 0.001
Ethnicity								
Malay	1.000				1.000			
Chinese	0.645	0.55	0.76	< 0.001	0.760	0.63	0.92	0.004
Indian	0.629	0.53	0.76	< 0.001	0.616	0.51	0.75	< 0.001
Other Bumiputeras	1.092	0.92	1.30	0.318	0.881	0.73	1.06	0.177
Others	0.997	0.76	1.31	0.982	0.994	0.72	1.38	0.970
Parent’s Marital Status								
Married	1.000				1.000			
Separated/Widow/Widower	1.542	1.43	1.67	< 0.001	1.189	1.08	1.32	0.001
Loneliness								
Yes	11.128	10.22	12.11	< 0.001	6.523	5.90	7.21	< 0.001
No	1.000				1.000			
Has close friend								
Yes	1.000				1.000			
No	3.321	2.88	3.82	< 0.001	2.045	1.70	2.47	< 0.001
Unable to sleep due to worry								
Yes	10.312	9.32	11.41	< 0.001	5.925	5.26	6.67	< 0.001
No	1.000				1.000			
Bullied								
Yes	3.231	2.90	3.60	< 0.001	2.847	2.46	3.30	< 0.001
No	1.000				1.000			
Lack of Parental Bonding								
Yes	2.053	1.91	2.20	< 0.001	1.403	1.27	1.55	< 0.001
No	1.000				1.000			
Lack of Parental Connectedness								
Yes	2.250	2.04	2.49	< 0.001	1.581	1.39	1.79	< 0.001
No	1.000				1.000			
Truancy								
Yes	1.777	1.66	1.91	< 0.001	1.430	1.31	1.56	< 0.001
No	1.000				1.000			

Analysis was done using complex sample logistic regression analysis. Classification Table (82.1%) were used to check model fitness

## Discussion

This investigation delves into the prevalence of depressive symptoms among secondary school students, revealing a prevalence of 26.9%, which is notably lower than the results obtained in a prior study conducted in Malaysia. The mentioned study involved 1800 Malaysian adolescents aged 13 to 17 years and reported a higher depressive symptoms prevalence of 32.7% [33]. It is noteworthy that both studies employed the same depressive symptoms assessment tool, the PHQ-9. However, the study by Normala et al. (2017) focused on 10 out of 37 randomly selected secondary schools in the Hulu Langat district of Selangor, providing a more localized perspective [33]. While the prevalence of depressive symptoms among Malaysian secondary school students stands at 26.9%, a look at our neighbours reveals some interesting similarities. In Indonesia, a study focusing on school-going adolescents using the PHQ-9 found a prevalence of 29.1% [34]. This figure is remarkably close to, and slightly higher than, Malaysia’s rate, suggesting that adolescents across Southeast Asia might face similar regional challenges or pressures. Similarly, our findings align closely with those from Bangladesh, where a study also using the PHQ-9 reported a prevalence of 26.5% for depressive symptoms among adolescents [35]. The consistent use of the PHQ-9 in these studies from Indonesia, Bangladesh, and Malaysia provides a solid foundation for these comparisons, hinting at shared underlying factors contributing to adolescent mental health in these regions. All comparison studies using the PHQ-9 that employ the same cut-off score classify respondents with scores of 10 and above as having depressive symptoms. In conclusion, the observed disparities in depressive symptoms prevalence among adolescents can be attributed to a myriad of factors, including demographic characteristics, methodological differences, and cultural, geographical, socioeconomic, and socio-political influences [9, 33–35]. A comprehensive understanding of these multifaceted factors is crucial for formulating effective health policies and programs tailored to enhance the mental health and well-being of adolescents across diverse countries. This recognition underscores the importance of context-specific approaches in addressing mental health challenges among adolescents on a global scale.

The outcomes derived from the multiple logistic regression analysis indicate a heightened susceptibility to depressive symptoms among female adolescents. Previous research has shown that elevated rates of depression in this population may be influenced by gender-specific factors, including differential parental treatment. In many cases, female adolescents are subjected to more stringent parental control compared to their male counterparts, which may contribute to heightened emotional distress [36]. Furthermore, societal norms and cultural expectations often place less value on the abilities and achievements of girls, potentially intensifying feelings of inadequacy or low self-esteem [36]. Empirical evidence has also demonstrated a clear gender disparity in the recognition and expression of depressive symptoms during adolescence. Specifically, male adolescents tend to suppress or underreport emotional distress, often refraining from acknowledging depressive symptomatology until it reaches an advanced or clinically significant threshold. This pattern is frequently attributed to socialized norms surrounding masculinity, which discourage emotional vulnerability and help-seeking behaviors. In contrast, female adolescents are generally more attuned to internal emotional states and are more likely to identify and disclose depressive symptoms at earlier stages of onset. This divergence in emotional expression and reporting may contribute to observed gender differences in the prevalence and detection of adolescent depression [37]. Our analysis confirms a statistically significant association between female gender and a greater likelihood of reporting depressive symptoms. Although our study does not directly investigate the mechanisms underlying this association, the observed gender difference aligns with established trends in the literature. Based on our findings, it is important for school-based mental health programs to adopt gender-sensitive approaches, ensuring that support systems are tailored to the specific needs of female adolescents, who may be more vulnerable to depressive symptoms. Future research should aim to explore the social and psychological factors contributing to this disparity to inform more targeted and effective intervention strategies.

In our study, we found that Chinese and Indian adolescents are less likely to experience depressive symptoms compared to their Malay counterparts. This finding contrasts with earlier research, which suggested that individuals of Chinese and Indian descent might be more susceptible to depression [3]. That earlier study utilized data from the Malaysia Global School-based Health Survey (GSHS) and the Mental Health Survey, both conducted as part of the 2012 National School-based Health Survey in Malaysia, involving 25,507 school-going adolescents [3]. One possible reason for this difference is the cultural strengths found in the Chinese and Indian communities in Malaysia. These groups often value strong family bonds and close community ties, which can offer important emotional support and stability for teenagers. In these cultures, family support is a big part of daily life and plays an important role in helping young people deal with stress, anxiety, and emotional problems. Chinese and Indian families also tend to focus on doing well in school and follow group-focused (collectivist) values, where the needs of the family or community come before individual desires. These values can give teens a clear structure, set expectations, and help them feel a strong sense of identity and purpose, things that can protect them from depression [38, 39]. Feeling like they belong and are important to their family or community can make teens feel more supported and less alone. Also, traditional practices like meditation, prayer, and attending religious or cultural events, common in Chinese and Indian cultures can help them manage stress and keep their emotions balanced, which may reduce the chances of depression [39]. In short, strong family relationships, shared community values, focus on education, and regular involvement in cultural traditions may help build emotional strength. In Malaysia, this may explain why Chinese and Indian adolescents are less likely to experience depression compared to Malay adolescents.

Furthermore, ongoing research clarifies that older adolescents are at an increased risk of experiencing depressive symptoms, corroborating the findings from a study undertaken by Xu H et al. [40]. This indicates that as adolescents progress in age, they encounter a heightened vulnerability to the onset of depressive symptoms. Numerous contributing factors may underpin this trend, encompassing the physiological, emotional, and social transformations inherent to adolescence, intensified academic pressures, evolving family dynamics, and the heightened emotional awareness characteristic of older teenagers [41–43]. A comprehensive understanding of these intricacies is essential for the development of effective strategies aimed at addressing the mental well-being of older adolescents.

The recent investigation reveals a noteworthy association between adolescents experiencing parental separation, widowhood, or being raised by widowers and an increased susceptibility to depressive symptoms. This observation diverges from a prior study conducted in Nepal involving 312 randomly selected higher secondary school students, which found no substantial link between the marital status of parents and the occurrence of depressive symptoms among students [44]. Numerous studies consistently affirm that the marital status of parents and the quality of relationships between parents and adolescents play pivotal roles in the psychological well-being of adolescents. A meta-analysis conducted by Amato and Keith [45] delving into the effects of parental separation on the adaptation and mental health of children concluded that children of divorced parents exhibited a slightly lower level of psychological well-being during adolescence compared to those with parents who remained together, although the difference was relatively modest. Furthermore, various research studies indicate that adolescents with separated parents statistically experienced more mental problems than those living in complete, intact families [46]. Research contributed to this understanding, highlighting that parental divorce significantly impacted the psychological stability of their children, particularly during adolescence, leading to negative consequences, particularly in terms of psychosocial impairment, such as frequent depressive moods [47, 48]. In summary, the collective evidence suggests a complex relationship between parental marital status and the mental health outcomes of adolescents, with a general trend pointing towards a higher likelihood of mental health challenges in those from separated or divorced families.

Loneliness and depressive symptoms are intricate emotional conditions that can significantly affect a person’s overall well-being. Our investigation disclosed that adolescents experiencing loneliness over the previous 12 months exhibited a higher likelihood of experiencing depressive symptoms. This observation aligns with the conclusions drawn by other researchers, highlighting a consistent correlation between loneliness and depressive symptoms in adolescents [16, 49]. Loneliness commonly stems from a lack of meaningful connections with others or limited social interaction. It’s a personal sense of being isolated and disconnected from people, often resulting in feelings of emptiness or being alone [16]. This pervasive sentiment transcends age, impacting individuals across the lifespan, including children and adolescents [50]. In contrast, depression is a more intense and pervasive mental health issue characterized by a consistently low mood, feelings of sadness, and a general lack of interest or satisfaction in various aspects of everyday life. Depression impacts not only emotional well-being but also cognitive and physical functioning. Those experiencing depression may find it hard to experience joy or fulfilment from activities they once enjoyed and may grapple with a pervasive sense of hopelessness [16]. Interestingly, there exists a reciprocal relationship between loneliness and depressive disorders. In individuals dealing with depression, loneliness frequently emerges as a concurrent symptom. The profound emotional and psychological impact of depression can lead to withdrawing socially and struggling to form or maintain interpersonal connections. Consequently, individuals with depressive disorders may find themselves facing an intensified sense of loneliness [16]. The link between depressive disorders and loneliness emphasizes the interconnected nature of mental health. Addressing one aspect, such as managing depression symptoms, may positively impact feelings of loneliness and vice versa. Recognizing and comprehending these dynamics is essential for developing comprehensive approaches to support individuals grappling with these challenging emotional states [16].

Within the school environment, the prevalence of bullying among students has emerged as a significant concern. Research findings indicate that, on a general scale, 15–30% of adolescents have experienced being bullied, although estimates vary based on the type of bullying victimization, geographic location, and the definition and measurement criteria for bullying [51, 52]. Notably, our survey revealed that adolescents who had experienced bullying at school within the past 30 days were nearly three times more likely to exhibit depressive symptoms. These results align with previous studies, highlighting a consistent correlation between bullying experiences and depressive symptoms [15, 53]. A theoretical framework suggests that challenging life experiences, such as being bullied, can induce various changes in both the cognitive and physiological aspects of individuals. Further studies have shown that individuals who have been bullied are more likely to report mental health issues, such as anxiety and depression [54, 55]. Longitudinal studies spanning extended periods consistently demonstrate these connections, indicating that being bullied is linked to enduring mental health problems over time [56, 57]. Additionally, research suggests that the duration of bullying experiences is proportional to the severity of subsequent mental health struggles. In simpler terms, the longer someone is subjected to bullying, the more pronounced the impact on their mental well-being becomes [58]. This emphasizes the importance for teachers and parents to be aware that prolonged exposure to bullying can significantly affect a student’s mental health. In light of these findings, it becomes imperative for schools to place increased emphasis on bullying prevention and the overall improvement of the school climate. Creating an environment that actively discourages and addresses bullying can play a crucial role in reducing depressive symptom levels among students. This underscores the collective responsibility of educators, parents, and administrators to foster a safe and supportive atmosphere within schools, thereby contributing to the overall well-being of students.

In both clinical [59] and community-based samples [60] of children and adolescents, disrupted sleep and depression frequently occur together, with prevalence rates varying considerably across different studies [61]. In this current study, there was a notable association between adolescents who experienced sleep difficulties and the presence of depression symptoms. There exists a fundamental intertwining relationship between sleep problems and depression, where disruptions in sleep not only align with an increased risk of encountering depression but are also recognized as secondary symptoms of the depressive condition. This implies that challenges in achieving quality sleep can both contribute to and manifest as symptoms of depression. The current body of evidence indicates that this relationship is bidirectional, signifying a mutual influence between sleep disturbances and depression [62]. However, drawing conclusive insights into the cause-and-effect dynamics between sleep disturbance and depression necessitates additional research. While existing findings offer valuable indications, further investigations are crucial to unravel the complexities of how disturbances in sleep might contribute to the onset of depression and, conversely, how depressive states might worsen sleep-related issues. In essence, a more thorough understanding of this intricate relationship requires ongoing exploration and in-depth analysis.

The current study identified a significant association between the absence of close friendships and depression symptoms. Additionally, regarding the home environment factor, adolescents who reported a lack of parental bonding and connectedness had higher odds of reporting depressive symptoms compared to their counterparts who experienced parental bonding and connectedness. Aligning with the World Health Organization (WHO), a growing body of evidence suggests that both parent involvement and the presence of close friendships play protective roles against mental health problems among adolescents [63]. Various studies have consistently highlighted the positive impact of parental involvement and strong friendships on the mental well-being of adolescents [64–67]. Research indicates that adolescents with close friendships are more likely to report better physical and mental health outcomes, emphasizing the role of social connections in overall well-being [66]. Conversely, those with insecure attachments or a lack of close ties are at a higher risk of developing depression [18, 19]. This underscores the importance of both family and peer relationships in shaping the mental health landscape of adolescents. In essence, the findings of the study underscore the interconnectedness of social relationships and mental health outcomes among adolescents. The presence of close friendships and positive parental bonding emerges as protective factors, while the absence of these connections may contribute to an increased vulnerability to depression. Recognizing these dynamics is crucial for developing targeted interventions and support systems aimed at fostering healthy social connections for adolescents, both within their families and peer groups.

The significance of consistent school attendance has been underscored by numerous studies, which consistently highlight the association between prolonged absence from school and poorer academic achievement [68, 69]. Furthermore, research suggests that persistent school absenteeism not only predicts early school leaving but also correlates with later unemployment and health problems, forming a trajectory with lasting implications [70, 71]. Our study found a strong association between truancy and depressive symptoms in adolescents. This shows us that skipping school is not just about attendance; it has wider implications for a young person’s mental well-being. It is a complex interplay between staying engaged in school and their psychological health. To truly understand truancy, we need to consider all the angles. It’s often influenced by a mix of factors, including a student’s family life, their community environment, and any existing mental health challenges they might be facing [71, 72]. For interventions and support to be effective, they must consider this complexity and be tailored to the unique situations of adolescents struggling with irregular school attendance. In summary, the findings of this study not only reinforce the importance of regular school attendance but also draw attention to the intricate relationship between truancy and mental health, urging educators, parents, and policymakers to adopt comprehensive strategies that address the underlying causes and provide appropriate support for adolescents facing challenges in their school attendance.

### Limitation

Our study has several limitations. Firstly, the data obtained in this study were self-reported by the participants, potentially incurring some recall or reporting bias. Consequently, there was no verification of diagnoses by healthcare providers. Secondly, this study is cross-sectional, thereby limiting the determination of the temporal relationship between the studied independent variables and depressive symptoms in order to establish the cause-effect relationship. Another limitation is the possibility of social desirability bias, which could cause adolescents to underreport their depressive symptoms, potentially leading to an underestimation of the true prevalence within this population. However, it should be noted that this study utilized extensive secondary data representative of adolescent populations in Malaysia. We recognize that single-item measures may have limitations in terms of reliability and depth; however, prior studies have demonstrated their effectiveness in capturing essential aspects of these constructs, especially within large-scale epidemiological surveys. Moreover, these items have been consistently employed in previous NHMS cycles (2012, 2017, and 2022), enabling comparisons over time. This limitation has been acknowledged, with an emphasis on the need for future research to utilize validated multi-item scales for a more comprehensive assessment.

## Conclusion

Depressive symptoms pose a significant concern among adolescents attending schools in Malaysia, with a prevalence rate of one in four. Several associated factors have been identified, including female gender, older age, parental separation or widowhood, loneliness, lack of close friends, sleep difficulties, bullying, truancy, and poor parental bonding and connectedness. In contrast, adolescents of Chinese and Indian ethnicity were less likely to experience depressive symptoms. In light of these findings, there is an urgent need to prioritize the development of targeted prevention and intervention programs specifically tailored for adolescents at risk. By focusing on these identified correlates through carefully designed initiatives, it is possible to reduce the prevalence of depressive symptoms and enhance the overall mental health and well-being of this vulnerable group. Adopting a strategic approach to mental health support, guided by a comprehensive understanding of the contributing factors, can significantly contribute to cultivating a healthier and more resilient adolescent population within the Malaysian school system.

## Data Availability

The datasets generated and/or analysed during the current study are not publicly available due to data protection purposes but are available from the corresponding author on reasonable request.
